# High-Power Laser Deposition of Chitosan Polymers: Medical and Environmental Applications

**DOI:** 10.3390/polym14081537

**Published:** 2022-04-10

**Authors:** Georgiana Cocean, Alexandru Cocean, Cristina Postolachi, Silvia Garofalide, Georgiana Bulai, Bogdanel Silvestru Munteanu, Nicanor Cimpoesu, Iuliana Cocean, Silviu Gurlui

**Affiliations:** 1 Faculty of Physics, Alexandru Ioan Cuza University of Iasi, 11 Carol I Bld., 700506 Iasi, Romania; cocean.georgiana@yahoo.com (G.C.); alexcocean@yahoo.com (A.C.); tina.postolaki@gmail.com (C.P.); silvia.garofalide90@gmail.com (S.G.); muntb@uaic.ro (B.S.M.); nicanor.cimpoesu@tuiasi.ro (N.C.); 2 Rehabilitation Hospital Borsa, 1 Floare de Colt Street, 435200 Borsa, Romania; 3 Integrated Center of Environmental Science Studies in the North-Eastern Development Region (CERNESIM), Department of Exact and Natural Sciences, Institute of Interdisciplinary Research, Alexandru Ioan Cuza University of Iasi, 700506 Iasi, Romania; georgiana.bulai@uaic.ro; 4 Faculty of Material Science and Engineering, Gheorghe Asachi Technical University of Iasi, 59A Mangeron Bld., 700050 Iasi, Romania

**Keywords:** laser-induced hydrolysis, hemostatic, biocomposite, medical devices, transdermal patches, chitin deacetylation, chitosan

## Abstract

High-power laser irradiation interaction with natural polymers in biocomposites and Laser-Induced Chitin Deacetylation (LICD) was studied in this work, in order to produce thin films consisting of chitosan composite. The new method can lead to a cutting-edge technology, as a response to the concern regarding the accumulation of “natural biological waste” and its use. The process consists of high-power laser irradiation applied on oyster shells as the target and deposition of the ablated material on different substrates. The obtained thin films we analyzed by FTIR, UV-VIS and LIF spectroscopy, as well as SEM-EDS and AFM. All the results indicated that chitin was extracted from the shell composite material and converted to chitosan by deacetylation. It was, thus, evidenced that chemical transformation in the chitin polymer side-chain occurs during laser irradiation of the oyster shell and in the resulted plasma plume of ablation. The numerical simulation in COMSOL performed for this study anticipates and confirms the experimental results of chitin deacetylation, also providing information about the conditions required for the physico-chemical processes involved. The high sorption properties of the thin films obtained by a LICD procedure is evidenced in the study. This quality suggests that they should be used in transdermal patch construction due to the known hemostatic and antibacterial effects of chitosan. The resulting composite materials, consisting of the chitosan thin films deposited on hemp fabric, are also suitable for micro-filters in water decontamination or in other filtering processes.

## 1. Introduction

Chitosan has been continuously studied due to its properties, which make it a material suitable for a large number of uses in medicine, such as wound patches with a hemostatic effect [1], drug delivery devices [2,3], in orthopedic tissue engineering due to a proper combination for biocompatibility, antibacterial properties and its suitable quality for cell growth [4,5,6]. Other chitosan uses refer to food supplements and as an agent for food preserving based on its antioxidant properties [7]. Environmental cleaning procedures have benefited from chitosan’s property to adsorb heavy metals on its carbonyl (C=O) groups, providing a “biosorbent” material for water decontamination [8,9,10,11,12] or for urea adsorption [13]. The high calcium carbonate content included in the composite structure of crustacean shells led to their use in construction industry as cement and for artificial stone fabrication [14,15].

An important aspect in environmental issues is the accumulation of “natural biological waste”. Such pollutants are neither measured nor regulated for the simple reason that they are generated naturally, and the only control is either the destruction of such residues or their use. The accumulation of “natural biological waste” occurs because there are certain biological “products” that decompose over very long periods of time, within thousands of years. Sometimes, only partial degradation occurs and not decomposition to chemical compounds that could be reintegrated into the natural circuit by themselves. This category of “natural biological waste” includes oyster shells, but it is not limited to them. There are already concerns about the accumulation of shells in some areas, some coming from the food industry, being perceived as industrial waste, and others are deposited as sediments on the bottom of seas and oceans or on the shores, sometimes causing serious problems [14,15].

Until now, chitosan has only been produced by the classical method, which consists of chemical treatments of demineralization and deproteinization to remove the impurities and to separate the chitin, which is deacetylated after that into chitosan by alkaline hydrolysis at high temperature [6,11,13,16,17,18,19]. Once obtained by the classical method, chitosan use was reported in further procedures where functional groups were added [13,20] or to produce chitosan composites [12] and chitosan nanocomposite with copper nanoparticles [21], as well as colloids with chitosan/lecithin nanoparticles [2], or in other processes to produce new materials suitable for advanced applications in medicine, optoelectronics and other domains.

Laser-induced chitin deacetylation (LICD) into chitosan, proposed in this paper, is a new method to produce chitosan thin films direct from oyster shells. It is important to develop this cutting-edge technology to produce chitosan thin films. The method is designed to be performed directly on the raw oyster shell, which is used as target without previous treatments, preparation and/or material(s) extraction. A large number of domains will benefit from this for experimental research and functional devices.

The thin films of chitosan obtained on different substrates result in composite materials when deposited on hemp fabric for further applications, such as medical transdermal patches, micro-filters for water decontamination or optical devices when deposited on glass or quartz slabs. Other substrates for depositing chitosan thin films may expand the area of applications.

Considering the advantages that such a method may bring to different technologies and industries, where chitosan thin films need to be produced, we are exposing further the method of work, reporting the results, analysis and interpretation of those results, indicating in the conclusions some of their possible uses and the need to continue studies on this topic.

## 2. Materials and Methods

Pulsed laser deposition technic (PLD) was performed on both glass slab and hemp twill fabric in order to study the physico-chemical properties of the obtained thin films, as well as the physico-chemical processes and mechanisms developed under laser interaction with the natural biocomposite of oyster shells that led to obtaining the thin films of a certain chemical composition. Neither preliminary preparation nor chemical treatments were performed on the oyster shells used as targets in pursuing the purpose of the study to find new uses for such raw materials, and also to investigate the natural biocomposite behavior as a whole and by its components under laser irradiation and the results of the ablation and deposition process.

The investigation of the new procedure that we purpose herein was assisted and completed by COMSOL numerical simulation with finite element method (FEM) of heat effects induced by the laser beam on the composite structure where chitosan and calcium carbonate are considered, in thermal interaction, as being the main components of the oyster shell.

**Figure 1:**
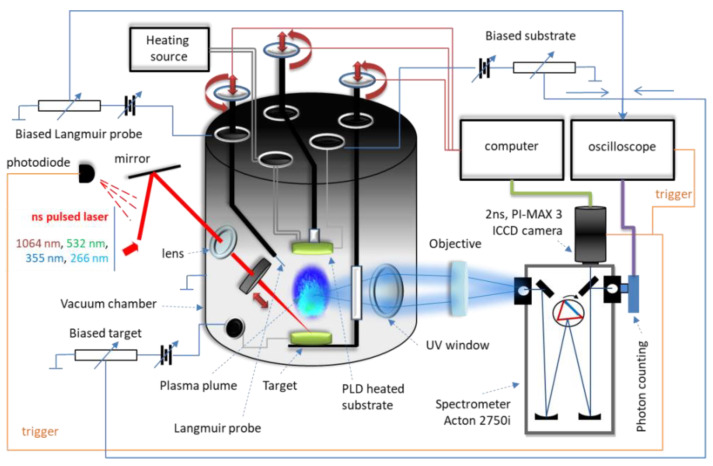
The experimental installation with deposition chamber.

For oyster shell irradiation, the YG 981E/IR-10 laser system (Quantel, Les Ulis, France) in the installation of Figure 1 was used with the following parameters: τ = 10 ns pulse width, λ = 532 nm wavelength, α = 45° incident angle, ν = 10 Hz pulse repetition time, r = 336 μm spot radius, F = 25 J/cm^2^ fluence, d = 3.5 cm distance between target and support.

The laser beam wavelength of 532 nm (in visible) was chosen because CaCO_3_, one of the main components in oyster shell [16,18], does not absorb the laser beam of 532 nm [22,23,24] as opposed to chitin, the component of interest for deposition and which will absorb the laser radiation of 532 nm [25]. Therefore, a high ablation rate of chitin was expected. The process is a selective extraction of one component of the composite material due to the different response of the components to the laser irradiation. Further, the 532 nm wavelength is preferred, being expected to be more suitable for a less aggressive interaction with the polymerized structures of the components in the oyster shell, same reason as reported by I. Cocean et al., 2019 and A. Cocean et al., 2021 on pulsed laser extraction and deposition of alpha-keratin [26] and of curcuminoids, respectively [27].

The analyses consisting of Fourier-transform infrared (FTIR) spectroscopy, laser-induced fluorescence (LIF) spectroscopy and Energy Dispersive X-ray coupled with Scanning Electron Microscope (SEM-EDS) were performed for the oyster shell used as target and for the deposited thin film. Atomic Force Microscopy (AFM) analysis provides information on the chitosan thin film morphology and topography. UV-Vis spectrum of the thin film obtained is also presented.

## 3. Results and Discussion

### 3.1. Numerical Simulation in COMSOL

**Figure 2:**
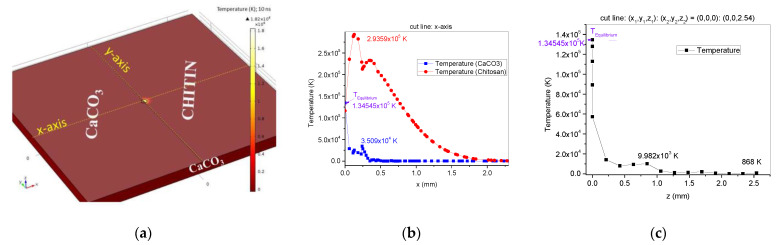
COMSOL simulation of laser-irradiated nonhomogeneous target with CaCO_3_ and chitin components: target components and temperature (K) developed on its surface 10 ns after laser pulse ignition (**a**); phase change diagram T(x), 10 ns after laser pulse ignition (**b**); phase change diagram T(z), 10 ns after laser pulse ignition (**c**).

For a better understanding and for receiving complementary information that will be used in the results interpretation for method validation, a simulation in COMSOL 5.6-01 version of the software (COMSOL AB, Stockholm, Sweden) was conducted, as to the thermal effect of the laser beam and heat transfer between the main components (CaCO_3_ and chitin) of the oyster shell biocomposite. In this experiment, we aimed to obtain chitosan thin film using a new method, which involves pulsed laser irradiation applied to an oyster shell. For this purpose, in the first stage, the thermal process during laser beam interaction with the target, which contributes to the chitin laser-induced deacetylation process, was anticipated based on a COMSOL simulation. Following the oyster shell components, the model in COMSOL was built in a rectangular 3D geometry of nonhomogeneous composition, consisting of calcium carbonate and chitin (Figure 2a), similar to A. Cocean et al., 2017 [22]. The area of interest in the simulation is the irradiated area in which the thermal effects occur. The heating effects can be observed in the 3D plot generated in COMSOL (Figure 2a) for temperature magnitude and distribution on the surface (irradiated side of the surface), 10 ns after laser pulse ignition. This plot offers primary information that the laser parameters used in the simulation induce the heating effects, which can lead to ablation. In order to estimate the areas subject to ablation, analysis of the phase transformations along the *x*-axis (cut line covering both components: calcium carbonate and chitin) and along the *z*-axis was performed.

The Heat Transfer in Solids module in COMSOL was completed with the numerical model, settings and boundary conditions, as per A. Cocean et al., 2017 [22,23].

The power of the laser beam (532 nm) was absorbed by the chitin component of the target and then transferred in volume. Calcium carbonate does not absorb the 532 nm laser radiation. As a result of the laser irradiation of the oyster shell target, heating effects induced directly by the laser radiation absorbed by chitin, and indirectly, by thermal diffusion from chitin to the calcium carbonate component, take place. The mathematical and theoretical model used is the one presented by A. Cocean et al., 2017 [22,23]. It is evidenced in the results of the simulation that both calcium carbonate on the upper surface, which is the side exposed to the laser irradiation (Figure 2a,b), and calcium carbonate positioned under the chitin-irradiated area (Figure 2a,c), may develop a high temperature of 10^5^ and 10^4^ K order, due to the heat diffusion process, tending to thermal equilibrium at the boundaries between the component materials of the oyster shell biocomposite (CaCO_3_ and chitin simulated herein). Calcium carbonate versus chitin ablation ratio of about 1:5 was calculated based on the average areas where conditions of temperature for melting and evaporation are met. The measured dimension of the damaged area along the *x*-axis, on the top surface, was found to be 0.4 mm, while for chitosan, a damage threshold of 2.19 mm was measured (Figure 2b). In the depth of the target, along the *z*-axis, the calcium carbonate CaCO_3_ component placed in the target volume, on the spot center direction (Figure 2a), is also affected by the heat diffusion process and the simulation shows significant thermal effects induced in calcium carbonate, over a distance of 0.42 mm.

Melting point was considered in the phase change diagrams of Figure 2b,c as the starting point for ablation. However, the ablation temperatures for both studied components coincide with their chemical transformations, meaning CaCO_3_ decomposition into CaO and CO_2_ and chitin deacetylation into chitosan and acetate, respectively. Furthermore, the high temperatures (T > 10^4^ K) that the simulation indicates as developing on the calcium carbonate component during the heat diffusion process provide the conditions to generate plasma, and CaO will result into ions of calcium and oxygen. The calcium ions can interact with the acetate ions resulted from chitin hydrolysis, forming calcium acetate ${\left(CH_{3}\;COO^{-}\right)}_{2}Ca^{2+}$. The results of the simulation were confirmed in the FTIR compared analysis of the oyster shell material and of the thin film obtained with the high-power laser deposition method.

### 3.2. Fourier-Transform Infrared Spectroscopy Analysis of Oyster Shell and Deposited Thin Film

In this experiment, chitin from an oyster shell was intended to be transformed and extracted as chitosan based on a laser-induced process.

**Figure 3:**
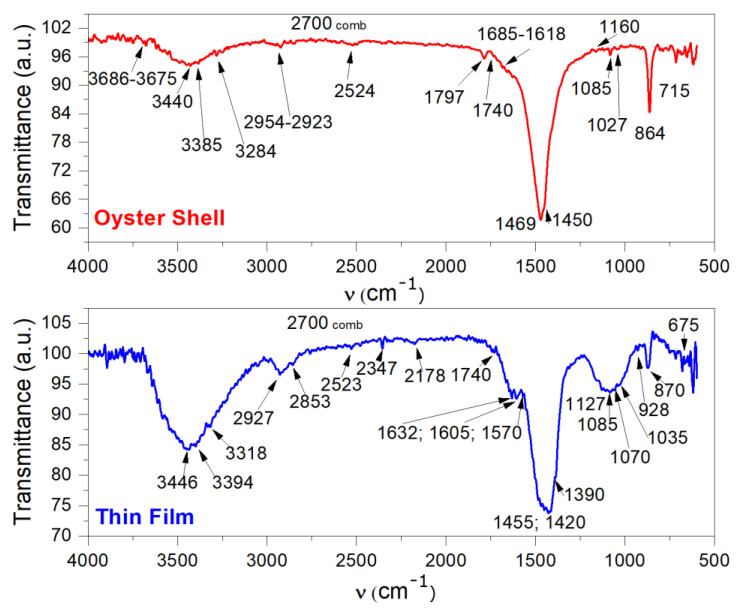
FTIR spectra of Oyster Shell natural biocomposite and thin film obtained with LICD method.

**Figure 4:**
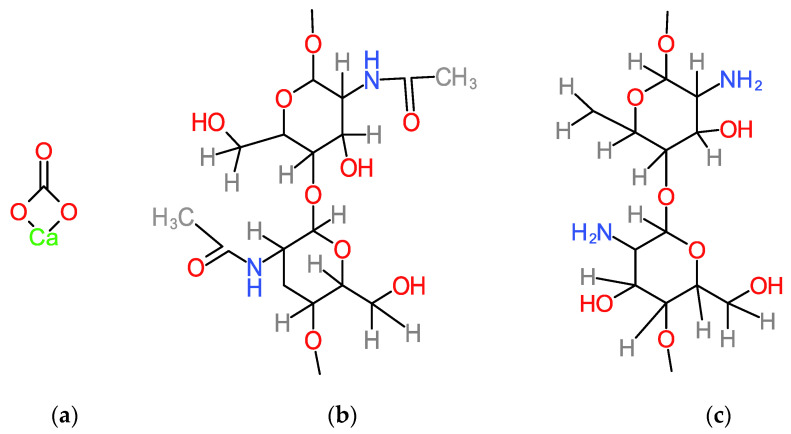
Calcium carbonate, CaCO_3_, structural formula (**a**); chitin, β-(1,4)-*N*-acetylglucosamine, structural formula (**b**) and chitosan, β-(1,4)-d-glucosamine, structural formula (**c**).

In the FTIR analysis (Figure 3, Oyster Shell spectrum), the main components in oyster shell, calcium carbonate and chitin (Figure 4a,b), as well as remnant water and residual proteins are evidenced by specific bands corresponding to their functional groups (Table 1). The laser-induced chitin deacetylation process is evidenced by changes in vibrational modes in the two spectra. Thus, the amide groups in the oyster shell become amino groups in the thin film and, at the same time, in the thin film spectrum, the specific vibrations of acetates are identified.

The detailed analysis of the FTIR spectra is presented below.

Table 2 presents the FTIR spectrum bands in Figure 3 (Thin Film) of the thin film obtained, identifying the chemical species that resulted from 532 nm, 25 J/cm^2^, 10 ns pulsed laser irradiation as being chitosan (chitosan formula in Figure 4c), acetate, acetic acid and adsorbed CO and CO_2_ gas phase.

In Table 1 and Table 2, based on the FTIR spectra in Figure 3, the main changes in the two spectra provide the information that $NHCOCH_{3}$ groups from chitin were transformed into $NH_{2}$ groups. This shows that chitosan was produced, as a result of alkali hydrolysis or deacetylation. The changes in the two spectra consist of shifts from 3440 cm^−1^ and 3385 cm^−1^ vibrational bands of chitin −NH groups (Figure 3, Oyster Shell spectrum and Table 1) to 3446 cm^−1^ and 3394 cm^−1^ vibrational bands of chitosan –$NH_{2}$ groups (Figure 3—Thin film spectrum and Table 2). Vibrational deformation bands of chitin −NH groups (in the range of 1560–1510 cm^−1^), overlap in the 1469 cm^−1^ band (Figure 3—Oyster Shell spectrum and Table 1), while bands at 1632 cm^−1^, 1605 cm^−1^, 1570 cm^−1^ are assigned to the chitosan –$NH_{2}$ groups (Figure 3—Thin film spectrum and Table 2). Carbonyl of chitin amide groups, as multiple picks in a range between 1685 cm^−1^ and 1618 cm^−1^, are evidenced (Figure 3—Oyster Shell spectrum and Table 1). In Figure 3—Oyster Shell spectrum and Table 1—the very strong band at 1469 cm^−1^, together with bands at 864 cm^−1^ and 715 cm^−1^ for in plane and out of plane, respectively, deformations, indicate the chitin amide groups, while in Figure 3—Thin film spectrum and Table 2—the bands at 1455 cm^−1^ and 1420 cm^−1^ evidence acetate formation together with the bands at 3446 cm^−1^, 2523 cm^−1^, 1632 cm^−1^, 1570 cm^−1^ of stretching vibrations and the specific band at 928 cm^−1^ of deformation vibration of acetates (Figure 3—Thin film spectrum and Table 2). The very intense bands at 1469 cm^−1^ in the oyster shell and 1455 cm^−1^ in the thin film are also evidence of a crystalline state and the shift denotes a change in the chemical composition that may be assigned to chitin deacetylation into chitosan and to calcium carbonate transformation into calcium oxide.

However, not all acetyl amide groups from chitin were transformed into amino groups. Remnant acetyl amide groups could be in the thin film. Specific bands may still be present in the spectrum (Figure 3—Thin film spectrum and Table 2), overlapping in the 3446 cm^−1^ band.

The bands for the other main groups in chitosan, those which are the same as for chitin, are slightly shifted (Table 1 and Table 2 and Figure 3—Oyster Shell and Thin film spectra) due to the lack of the carbonyl group in chitosan that influences vibrations in chitin by partially moving the electrons toward the strong electronegative oxygen atom.

The FTIR spectrum of the thin film (Figure 3—Thin film spectra) shows bands assigned to the CO and CO_2_ adsorbed gas phase (resulted from calcium carbonate decomposition) at 2178 cm^−1^ and 2347 cm^−1^/675 cm^−1^, respectively. The band at 1740 cm^−1^ is assigned to formaldehyde, naturally produced in the cell metabolism of the oyster and adsorbed onto its shell. Naturally, chitin deacetylation is produced enzymatically by chitin deacetylase, an enzyme that catalyzes the chemical reaction of chitin with water H_2_O. For this reason, sometimes, an oyster shell may already contain chitosan if it was exposed for a long time to media favorable to specific fungi and insects. Depending on the deacetylation degree of the chitin, the specific bands may be shifted from one sample to another sample, resulting in differences in chitin and chitosan spectra.

FTIR spectra show that high-power laser irradiation has led to deacetylation of chitin into chitosan. Deacetylation is also confirmed by the acetate ions identified in the obtained thin layer spectrum. FTIR spectral analysis also indicates that calcium carbonate decomposed during pulsed laser deposition

### 3.3. Elemental Composition Analysis with Energy-Dispersive X-ray Spectroscopy Coupled with Scanning Electron Microscopy (SEM–EDX)

The elemental composition analysis was performed with Energy Dispersive X-ray Spectroscopy using a Scanning Electron Microscope (Vega Tescan LMH II, Brno, Czech Republic). The variation in the atomic percentage composition, from 49.41% at. Oxygen, 42.41% at. Carbon, 8.17% at. Calcium in the Oyster Shell to 70.92% at. Oxygen, 23.56% at. Carbon, 5.52% at. Calcium for the obtained thin film shows an increase in oxygen and a mitigation in the calcium atomic percentage. The mitigation in calcium atomic percentage in the thin film is in good accordance with the lower ablation rate of calcium carbonate, as anticipated in the COMSOL simulation. The increase in oxygen atomic percentage is in accordance with the anticipated process of calcium carbonate heating and decomposition, as described based on the COMSOL simulation and as validated by the FTIR spectrum.

**Figure 5:**
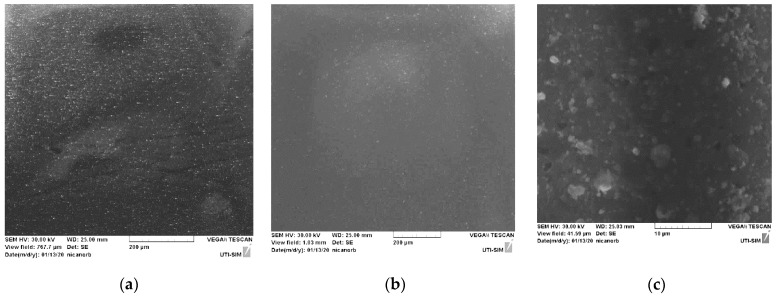
SEM images of oyster shell 250× magnified (**a**), PLD thin film 200× magnified (**b**) and PLD thin film 5k× magnified (**c**).

The images provided by the SEM analysis indicate the granular morphology of the obtained chitosan thin film (Figure 5b,c), similar to the morphological structure exhibited in the image of the oyster shell (Figure 5a). The elemental analysis indicates chitin deacetylation and calcium carbonate decomposition, as anticipated by the simulation in COMSOL and in accordance with FTIR analysis.

### 3.4. Laser-Induced Fluorescence

**Figure 6:**
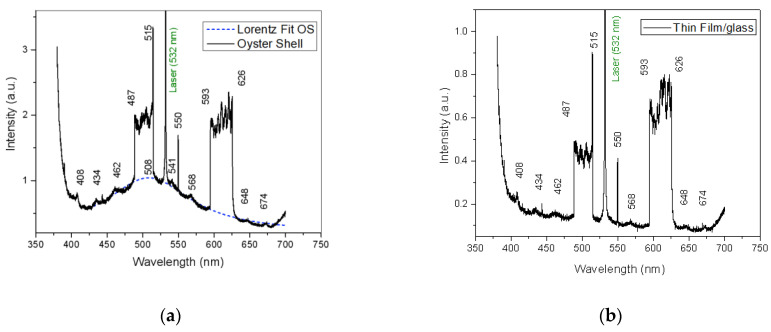
Laser-induced fluorescence spectra, 30 ns delay of the oyster shell used as target (**a**) and of the thin film deposited on glass slab (**b**).

As iterated before, the 532 nm laser wavelength was chosen because calcium carbonate does not absorb at this wavelength and to minimize calcium carbonate extraction. When performing laser-induced fluorescence (LIF) spectroscopy with 355 nm wavelength pulsed laser beam (10 ns pulse width and 10 Hz repetition rate) using 150 mJ laser energy on the Oyster Shell target and on the thin films deposited on hemp fabric and on glass, two enhancements, tower shaped, are noticed between the ranges of 487–515 nm and of 593–626 nm (Figure 6a), which have been assigned to chemical reactions, mainly the formation of radicals [26,27,36]. The source of hydroxyl radicals may be chitin and chitosan, as well as residual water in a dissociation process, under UV pulsed laser irradiation at a 355 nm wavelength. The oyster shell spectrum Lorenz fit curve in Figure 6a shows that its characteristic fluorescence is at 508 nm. This peak in the spectrum of the oyster shell target is no longer found in the spectrum of the thin film on glass (Figure 6b). That peak may be assigned to the amide structure of chitin as a fluorophore group in the oyster shell material. Specifically, fluorescence at 508 nm can be assigned to the carbonyl groups that are missing in the thin layer, which consists mostly of chitosan.

The emission at 462 nm is assigned to purified chitin autofluorescence in the oyster shell spectrum [37] and also to chitosan when excited at a 355 nm wavelength [38]. The peaks of fluorescent emission spectra at 408, 434, 568, 648 and 674 nm are specific for both the oyster shell used as the target in pulsed laser deposition and the thin film obtained.

They may denote chemical reactions, including the formation of excimers, such as acetate. In addition, the line at 550 nm in the fluorescence emission spectra indicates a mirrorless laser phenomenon, which could be in connection with the formation of excimers. Although excimer formation has already been reported in the scientific literature, including during fluorescence sensing, more investigation is still required, with regard to these processes [39,40,41].

LIF analysis evidenced a short fluorescence lifetime for all analyzed samples. The maximum fluorescence was noticed after 30 ns from laser irradiation for the thin films. Fluorescence was still observed after 50 ns for the oyster shell, but the spectrum was affected by severe noise.

The fluorescence of the chitosan thin film, obtained using the high-power irradiation method, is similar to that of the oyster shell used as the target in the process, except the peak at 508 nm, which is assigned to the acetyl group that was removed. This indicates that the thin film is the result of chitin deacetylation.

### 3.5. UV-VIS Thin Film Spectra Analysis

**Figure 7:**
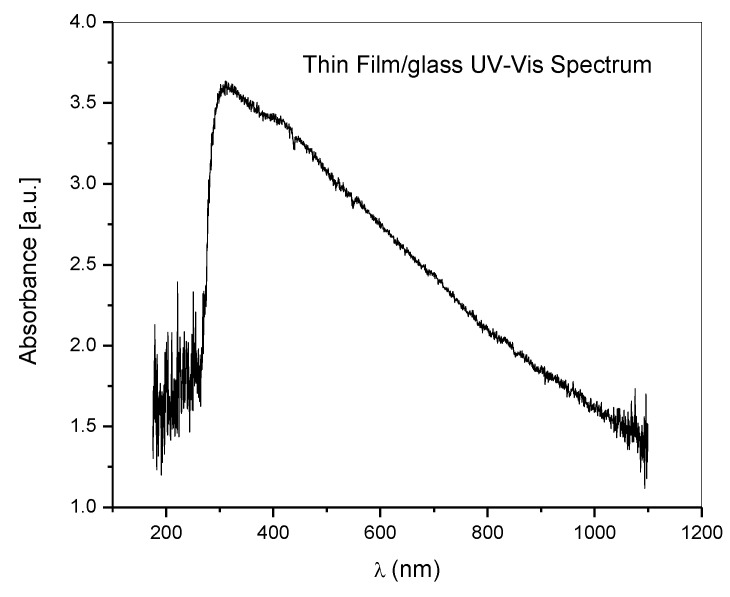
UV-VIS spectra of deposited thin film on glass.

The UV-VIS absorption spectrum of the sample in the 200–1100 nm wavelength range was recorded using a UV-VIS-NIR Ocean Optics (DT-MINI-2-GS, Ocean Insight, Rochester, NY, USA). Light Source and an Avantes spectrometer (AvaSpec 2048, Avantes, Apeldoorn, The Netherlands). The UV-VIS absorption spectrum of the thin film deposited on glass is presented in Figure 7. The results confirm that the thin film obtained with the method presented herein is chitosan.

The UV-VIS absorption spectrum is similar to those previously reported in papers for chitosan by the maximum absorption, around 300 nm [42,43]. A second peak is noticed between 400 and 600 nm, which is usually assigned to metallic nanoparticles. That could be the influence of calcium atoms and ions embedded into the chitosan thin film.

The results confirm that the thin film obtained with the method presented herein is chitosan.

### 3.6. Atomic Force Microscopy Thin Film Analysis

**Figure 8:**
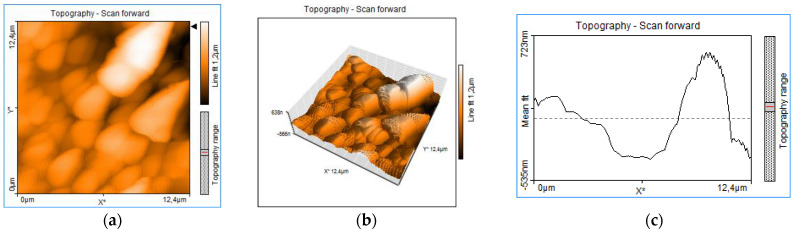
AFM thin film chitosan: 2D image 12 μm topography (**a**); 3D Topography 12 μm (**b**) and topography 3D line (**c**).

Atomic force microscopy (AFM) images, obtained with Nanosurf Easy Scan 2, Liestal, Switzerland, evidence the morphology of the chitosan thin film, with a granular distinct aspect. It is not only a surface roughness, but the grains are disposed in volume as distinct units, without forming a continuous, compact mass of material (Figure 8). Aggregated structures are indicated mostly by the 3D topography (Figure 8b) and by the 3D topographic line (Figure 8c), which exhibits overlapped peaks.

The images with scanning electron microscopy (SEM) in Figure 5 confirm the granular morphology with a disposition of certain uniformity on the surface of the target material (a), as well as on the surface of the thin layer (b and c). The images prove good conditions for an advanced sorption, as the available sorption surface related to volume is optimized due to the granular structure. This is a good quality for medical devices designed for sorption and desorption processes, such as transdermal patches. Similar morphological structures have been reported for the AFM analysis performed on chitosan-sulfated films, obtained through chemical procedures by Aleksandr S. Kazachenko et al., 2021 [44]. This similarity indicates that the morphology of the chitosan films is given by the chitosan polymer main chain, and it is independent of the method used.

### 3.7. Sorption Test of Thin Film of Chitosan

**Figure 9:**
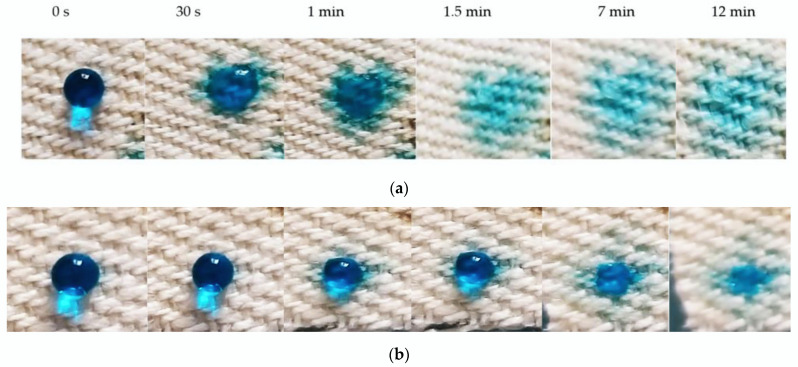
Sorption process of Reactive Blue 21 aqueous solution on Thin Film of Chitosan deposited by LICD from Oyster Shell on hemp twill fabric (**a**) and on hemp twill fabric itself (**b**).

The test with C.I. Reactive Blue 21 (Bezaktiv Turqoise Blue V-G from CHT Germany GmbH, Tübingen, Germany) droplets proved that the thin film obtained using the high-power laser deposition method on hemp fabric enhanced the sorption of aqueous solution, compared to the hemp fabric without the coating. The droplet started to be absorbed within a few seconds on the chitosan thin film, with complete absorption after only 1.5 min, compared to 12 min absorption time on the hemp fabric. On the chitosan thin film, the liquid was absorbed on an enlarged area compared to the droplet absorption on the hemp fabric (Figure 9). Thus, the droplets of RB21 aqueous solution, 10 mm in diameter, covered a surface up to 18 mm × 20 mm after only 1.5 min, when the sorption process in the thin film was complete. This indicates that the enhanced sorption is due to the chitosan thin film and to the hydrogen bonds and Van der Waals interactions between the amino groups in chitosan and hydroxyl groups in water. The droplet is, therefore, absorbed fast into the chitosan layer, and the liquid is kept at the surface of the fabric where the chitosan layer is disposed (Figure 9a). For the droplet placed on the hemp fabric without coating, the liquid is absorbed slowly. The absorption in the depth of the fabric and the sorption surface, in this case, is of about the same radius size as for the initial droplet (Figure 9b). This proves the high sorption properties of the thin film of the obtained chitosan.

Laser-induced chitin deacetylation, using oyster shell and, thus, extraction of chitosan that was deposited as thin film, is demonstrated by the changes noticed in the thin film spectrum bands compared to the FTIR oyster shell spectrum. FTIR spectroscopy shows vibrations specific to the oyster shell components chitin and calcium carbonates, but also remnant water in the shell and adsorbed formaldehyde. Vibration modes detected for the thin film are assigned to the chitosan chemical structure, proving chitin deacetylation. UV-Vis analysis of the thin film obtained by the LICD method, presented herein, resulted in a spectrum of similar characteristics as for the chitosan thin films obtained under the other methods. 

The COMSOL simulation provides valuable information on the heating effects, indicating thermal conditions for calcium carbonate decomposition. Furthermore, calcium carbonate in oyster shell composition provides the calcium ions for the acetate and also the alkali media required in the reaction of deacetylation. Granular topography of the thin layer indicates a form of composite-type aggregation, in which the components are physically individualized with the precise delimitation of each component, but compacted to form a common body. The structure looks similar to mortar. Calcium acetate and residual water, as well as traces of calcium carbonate and/or calcium oxide, may contribute to its compaction. Elemental composition analyzed with the SEM-EDX method and laser-induced fluorescence (LIF) also contributes to complete and to sustain the previous analysis, proving that deacetylation took place. Based on the LIF analysis, new information on excimer formation during UV pulsed laser irradiation is acquired. The excimer detected by LIF spectroscopy is assigned to the acetate dimer. On the laser-induced deacetylation, during high-power laser deposition, physico-chemical processes can be described by the following equations:

(1) $$CaCO_{3}\overset{t^{0}\geq 1114K}{\to }CaO+CO_{2}\;(Ca^{2+}O^{2-}\;\mathrm{in}\;\mathrm{plasma}\;\mathrm{of}\;\mathrm{ablation})$$

(2) $$CaO+H_{2}O\to Ca{\left(OH\right)}_{2}\;(\mathrm{alkali}\;\mathrm{media}\;\mathrm{in}\;\mathrm{plasma}\;\mathrm{of}\;\mathrm{ablation},\;Ca^{2+}2HO^{-})$$

(3) $$R–NHCOCH_{3}\overset{deacetylation}{\to }R-NH_{2}+CH_{3}COO^{-}\;(\mathrm{in}\;\mathrm{plasma}\;\mathrm{of}\;\mathrm{ablation})$$

(4) $$2CH_{3}COO^{-}+Ca^{2+}\to {\left(CH_{3}COO^{-}\right)}_{2}Ca^{2+}$$

Concluding the transformations during ablation and deposition, the following species are assessed at each step of the laser-induced chitin deacetylation process:

➢ **Oyster shell target**: $R–NHCOCH_{3}$; $CaCO_{3};H_{2}O$; adsorbed $H_{2}CO$
➢ **Ablation Plume**: $R–NH_{2}$; $Ca^{2+}O^{2-}$; Ca ; O; $O_{2}$; $OH^{-}$; $CO_{2}$; CO; $CH_{3}COO^{-}$; $H_{2}CO$
➢ **Thin film resulting from deposition on the substrate**: $R–NH_{2}$; ${\left(CH_{3}COO^{-}\right)}_{2}Ca^{2+}$; CaO adsorbed $CO_{2}$; adsorbed CO; adsorbed $H_{2}CO$

It is also evidenced that laser-induced deacetylation is laser-induced thermal hydrolysis. Calcium carbonate provides the alkali media under the laser thermal effects developed in the biocomposite material of the oyster shell. It is, therefore, evidenced with this study that the pulsed laser irradiation develops the same effect in chitin as in the alkaline hydrolysis, breaking the bonds between NH and C=OCH_3_ in the NHCOCH_3_ groups to form chitosan and acetate in the presence of the remnant water in the oyster shell. The physico-chemical process is evidenced both experimentally and by the simulation in COMSOL as laser-induced thermic hydrolysis, and in this specific case, as laser-induced chitin deacetylation. Although calcium carbonate does not absorb the 532 nm laser beam wavelength, the simulation in COMSOL anticipated that CaCO_3_ is ablated when in contact with laser-irradiated chitin due to heat diffusion phenomena. The elemental analysis confirms the results of the simulation through the calcium ions and atoms embedded in the thin layer. The computational models are important tools to set up parameters and conditions for experimental procedures and to complete the information on the processes and phenomena [45].

## 4. Conclusions

The method of laser-induced chitin deacetylation (LICD) has been proven to be very efficient and economically feasible to replace other methods, where the chitosan thin films are obtained from the chitosan powder produced by chemical means and procedures. This way, the three-step process (oyster shell—chitin separation through chemical processes; chitosan powder produced by chemical processes—deposition of the thin film of chitosan) becomes a one-step process (oyster shell—thin film of chitosan). The new method of laser-induced chitin deacetylation and deposition of thin layers with chitosan content (and the polymers obtained), direct from the oyster shell, is a potential method to produce hemostatic and antibacterial medical patches, and for drug delivery through the skin, due to the already proven chitosan properties. The increased sorption properties of the obtained chitosan thin film and its content in calcium make it suitable to be used in the fabrication of medical patches for wound dressing [46,47,48] and for transdermal patches [3]. Furthermore, the obtained thin film, by its sorption properties, is suitable for microfilter fabrication. Based on the fluorescent properties, evidenced by the LIF analysis, and due to its surface high sorption, the chitosan thin film obtained using the high-power laser deposition technique can be used to develop fluorescent chemosensor devices.

## Figures and Tables

**Table 1:** Oyster shell biocomposite material main components—the FTIR spectrum bands and interpretation.

Component	Functional Groups	Wavenumber (cm^−1^)	Observation and References
**CaCO_3_**	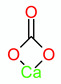	2524 weak	Chelates 2539 [28] 3200–2500 [29]
	*C=O*	1797 weak	*C=O* stretching vibration in calcium carbonate 1796 [30] 1785 [28] 1793 [31]
	C–O	1450 very strong, narrow	C–O stretching mode of carbonate; asymmetric stretch bands 1453 [32] 1425 [30] 1430 [28]
	$CO_{3}^{2-}$	1085 weak, narrow; 864 strong, narrow 715 weak	1085 symmetric stretch of carbonate ions $CO_{3}^{2-}$ and 864; 720 out-of-plane bending modes 1085; 876 [30] 1453; 873 [32] 877; 715 [28] 871; 711 [31]
	Lattice modes	1469 strong	Lattice modes [30]
Chitin	−OH	3686; 3675 sharp; 3440 broad, numerous bands	3650–3200 Free OH; 3550–3450 H–bonded OH; 3500–3200 polymer OH; broad, often numerous bands [29]
	−NH	3440; 3385 broad medium	3500–3100 amides with 3180: −NH stretch in secondary amides (2°); 1560–1510 (overlapped on the 1469 band: −NH deformation vibration in secondary amides (2°): *–NHC=O* stretch symmetric, H–bonded, also specific to polypeptides [29] 1567 [7]
	*–C=O*	1685–1618 multiple peaks	1685 *–C=O* stretching in primary amides *–NHC=O*; multiple picks assessed to different other components as impurities in the natural biocomposite [28] 1659 [7]
	*–NHC=O*	1469 very strong; 864; 715	1465 NH in plane deformation vibration ~800; ~700 NH out of plane deformation vibration [29]
	$-CH_{2}$	2954; 2923 Comb at ~2700	3000–2840 C–H stretching vibrations 2900–2850 cyclohexanes, weak, comb at ~2700 [29] 2924; 2853 [7]
	–OH (alcoholic)	3284 medium	Corresponds to 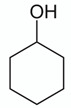 (3335, 1350 characteristic bands, the shift to 3284 due to the influence of the other functional groups, including to the oxygen contained in the oxane ring, and the 1350 band overlaps on the 1469 band) [29] 1378 [7]
	–C–OH	1085 weak	1075–1000 C–O stretching vibrations in $–CH_{2}–OH$ [28] 1084 [7]
	–CH–O–CH–	1160	1170–1115 –CH–O–CH– stretching symmetric vibrations bridge C–O–C [29] 1157 [7]
	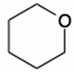 oxane (oxacyclohexane) in glucosamine ring	1085; 1027 864	1085; 1027 skeletal vibrations due to C–O stretching; ~1100 asymmetric; ~815 symmetric; shifts caused by the conjugated bonds in the chitin molecule, but also because of intra and intermolecular H-bondings and/or Van der Vaals interactions (among temporary dipoles and induced dipoles) [29] 1084; 892 [7] 1075; 1025 [12]
Remnant Water $\left(H_{2}O\right)$	–OH	3675 weak	–O–H free (water 3657) [29]
Formaldehyde	*H_2_C=O*	1740	1720/1500, bands for adsorbed formaldehyde [33] Adsorbed formaldehyde on the oyster shell may be the formaldehyde naturally produced by the oyster as its cells metabolism. The 1500 band in the spectrum overlaps on 1469 band.

**Table 2:** The FTIR spectrum bands and interpretation for the thin film obtained by LICD from raw oyster shell.

Component	Functional Groups	Wavenumber (cm^−1^)	Observations References
Chitosan	–OH	3446 strong, broad, numerous bands	3650–3200 Free OH; 3550–3450 H–bonded OH; 3500–3200 polymer OH; broad, often numerous bands; In the same range with $–NH_{2}$ [29] 3385.92 [8] 3478.68 [12] 3478.68 [12]
	$–NH_{2}$	3446; 3394 overlapped with the 3446 band; 1632, 1605; 1570 medium, partially overlaps with 1455–1420 bands	3650–3200; 3550–3450; 3500–3300; $–NH_{2}$ stretch in amines and 1650–1590 medium to weak for $–NH_{2}$ deformation vibration in amines: [29] 3385.92; 1635.60; 1545.60 [8] 3360–3440; 1629.85; 1529.55 [12] 1656.88; 1571.05 [12] 1661, 1559 [7]
	$–CH_{2}$	2927; 2853; comb at ~2700	3000–2840 C–H stretching vibrations 2900–2850 cyclohexanes, weak, comb at ~2700 [29] 2939.52 [8] 2924.13 [12] 2924; 2853 [7]
	–OH (alcoholic)	3318 medium	3318 band partially overlaps with the band at 3446 and a band at 1350 can be assessed as overlapping on the bands in the range 1455–1420 Corresponds to 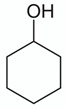 (3335, 1350 characteristic bands, the shift to higher wavenumbers is due to the influence of the other functional groups, including oxygen contained in the oxane ring) [29] 1387.7 [8] 1378.16 [12] 1377 [7]
	$–CH_{2}–OH$	1070 weak	1075–1000C–O stretching vibrations in $–CH_{2}–OH$ [29] 1075.33; 1025.18 [12]; 1072; 1022 [7]
	–CH–O–CH–	1127	1170–1115–CH–O–CH– stretching symmetric vibrations bridge C–O–C [29] 1025.18 [12] 1118 [7]
	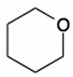 oxane (oxacyclohexane) in glucosamine ring	1085 870	skeletal vibrations due to C–O stretching; ~1100 asymmetric; ~815 symmetric; shifts caused by the conjugated bonds in the chitosan molecule, but also because of intra and intermolecular H-bonds and/or Van der Waals interactions (among temporary dipoles and induced dipoles) [29] 1096; 893.6 [8] 1075.33 [12] 1072: 816 [7] 1075; 1025 [12]
Acetates and acetic acid resulted from chitin deacetylation	–COOH (carboxyl)$–{\left(COO\right)}^{-}$ (carboxylate)	3446 strong, numerous bands; 2524 weak; 1632 medium; 1570 medium 1455; 1420 928 weak	3550–2500 variable intensity, “hairy beard” aspect of the band –COOH stretching vibrations; 1670–1650 *–C=O* stretching vibrations, intramolecular H-bonded; 1610–1550 $–{\left(COO\right)}^{-}$ stretching asymmetric vibrations with a 20 cm^−1^ shift; 1450–1400 $–{\left(COO\right)}^{-}$ stretching symmetric vibrations; ~925 acetates $–{\left(COO\right)}^{-}$ deformation vibration [29]
Adsorbed gas phase	Adsorbed CO	2178 weak	2200–2100 range, adsorbed CO on different metal oxydes (metals ionic state) used as catalysts [34] 2178 [34,35]
	Adsorbed CO_2_	2377 weak; 675 weak	2347 and 660 CO_2_ molecule adsorbed [33]
Formaldehyde	*H_2_C=O*	1740	1720/1500, specific bands for adsorbed formaldehyde [33] Adsorbed formaldehyde on the thin film could be sourced by the oyster shell, but may also be new molecules of formaldehyde resulted during laser induced deacetylation. The 1500 band in the spectrum overlaps on the 1455 band.

## Data Availability

Not applicable.
